# On Syphilitic Epilepsy

**Published:** 1871-09

**Authors:** 


### Books Reviewed.

---

*On Syphilitic Epilepsy.* By REUBEN A. VANCE, M.D.

Dr. Vance, who has devoted a considerable portion of his time to the observation of the disease of the nervous system, has written a very interesting paper on Syphilitic Epilepsy, which we receive in reprint from the American Journal of Syphilography and Dermatology. He says that in diagnosing this disease it is of the greatest importance to obtain a complete syphilitic history and to discern evidences of the persistence of the disease. The ophthalmascope is spoken of as being very valuable in this connection, for, through it we may observe the changes in the blood-supply of the retina indicating the brain-circulation, and also the effects of the remedies. The treatment must be specific, each case becoming a matter of special study. Several cases of epilepsy are given, caused by the influence of constitutional syphilis, which were successfully treated. From what he says in these cases we may infer that the disease, under proper treatment, readily yields, even if the patient has suffered for years from it.

